# Optimal Properties of Analog Perceptrons with Excitatory Weights

**DOI:** 10.1371/journal.pcbi.1002919

**Published:** 2013-02-21

**Authors:** Claudia Clopath, Nicolas Brunel

**Affiliations:** 1 Laboratory of Neurophysics and Physiology, CNRS and Université Paris Descartes, Paris, France; 2 Centre for Theoretical Neuroscience, Columbia University, New York, New York, United States of America; 3 Departments of Statistics and Neurobiology, University of Chicago, Chicago, Illinois, United States of America; Indiana University, United States of America

## Abstract

The cerebellum is a brain structure which has been traditionally devoted to supervised learning. According to this theory, plasticity at the Parallel Fiber (PF) to Purkinje Cell (PC) synapses is guided by the Climbing fibers (CF), which encode an ‘error signal’. Purkinje cells have thus been modeled as perceptrons, learning input/output binary associations. At maximal capacity, a perceptron with excitatory weights expresses a large fraction of zero-weight synapses, in agreement with experimental findings. However, numerous experiments indicate that the firing rate of Purkinje cells varies in an analog, not binary, manner. In this paper, we study the perceptron with analog inputs and outputs. We show that the optimal input has a sparse binary distribution, in good agreement with the burst firing of the Granule cells. In addition, we show that the weight distribution consists of a large fraction of silent synapses, as in previously studied binary perceptron models, and as seen experimentally.

## Author Summary

Learning properties of neuronal networks have been extensively studied using methods from statistical physics. However, most of these studies ignore a fundamental constraint in networks of real neurons: synapses are either excitatory or inhibitory, and cannot change sign during learning. Here, we characterize the optimal storage properties of an analog perceptron with excitatory synapses, as a simplified model for cerebellar Purkinje cells. The information storage capacity is shown to be optimized when inputs have a sparse binary distribution, while the weight distribution at maximal capacity consists of a large amount of zero-weight synapses. Both features are in agreement with electrophysiological data.

## Introduction

Purkinje cells (PCs) are the only outputs of the cerebellar cortex, a brain structure involved in motor learning. They receive a very large number (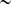
150,000) of excitatory synaptic inputs from Granule Cells (GCs) through parallel fibers (PFs), and a single very strong input from the inferior olive through climbing fibers (CFs).

Single PCs have long been considered as a neurobiological implementation of a perceptron [1], [2], the simplest feedforward network endowed with supervised learning [3], since CFs are thought to provide PCs with an error signal [4]. A perceptron learns associations between input patterns and a binary output that are imposed to it. Learning is due to synaptic modifications, under the control of an error signal. The learning capabilities of perceptrons have been extensively studied for unbiased [5], [6] as well as biased patterns [6], and for unconstrained synapses [5], [6]. In real neurons, synapses are either excitatory (glutamatergic synapses), or inhibitory (GABAergic synapses), depending on the identity of the pre-synaptic neurons (except during early development, when GABAergic synapses are initially excitatory and then become inhibitory). A multitude of experiments characterizing synaptic plasticity have shown that the strength, but not the sign, of a synapse can be modified by patterns of neuronal activity. This has led to the study of perceptrons with sign-constrained weights [7], [8], [9], [10]. In particular, Brunel et al. [10] showed that when synaptic weights are constrained to be excitatory (positive or zero), a perceptron at maximal capacity has a distribution of synaptic weights with two components: a finite fraction of zero-weight (‘silent’) synapses; and a truncated Gaussian distribution for the rest of the synapses. They further showed that this distribution is in striking agreement with experimental data [10].

Numerous experiments show however that in the course of specific motor tasks, the firing rate of Purkinje cell varies in an analog, not binary, fashion [11], [12], [13], [14]. We therefore set out to investigate the capacity and distribution of synaptic weights of a perceptron storing associations between analog inputs and outputs. More precisely, each input or output unit can take an analog value drawn from a distribution with a given mean and variance. We show that the optimal input distribution matches the firing pattern of the Granule cells, and weight distribution at maximal capacity reproduces the experimental Parallel Fiber to Purkinje cell synaptic weight distribution.

## Results

### The analog perceptron

The perceptron consists of 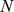 inputs and one output. Both inputs and outputs take continuous values. We require this perceptron to learn a set of 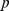 prescribed random input-output associations, where the inputs 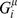 (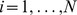, 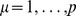) are drawn randomly and independently from a distribution 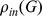, with mean 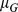 and standard deviation 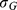 while the target outputs 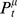 are drawn randomly and independently from a distribution 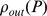 with mean 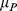 and standard deviation 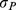. Note that since 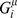 and 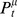 represent firing rates of input and output cells, respectively, they must be non-negative quantities. In particular, 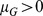, 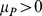 represent the mean firing rates of granule/Purkinje cell, respectively. The output of the perceptron when a pattern 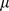 is presented in input is given by
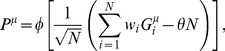
(1)where 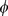 is a monotonically increasing static transfer function (f-I curve), 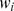 are the synaptic weights from input 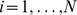, 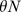 represents inhibitory inputs that cancel the leading order term in 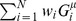 so that the argument of 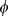 is of order 1. In Purkinje cells, these inhibitory inputs are provided by interneurons of the molecular layer. The goal of perceptron learning is to find a set of synaptic weights 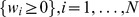 for which 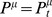 for all 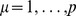.

We focus for simplicity on a linear transfer function 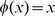, but our results can be applied to arbitrary invertible transfer functions 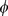. Indeed, the problem of learning associations (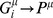) in a perceptron with an arbitrary invertible transfer function 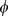 is equivalent to the problem of learning (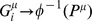) in a linear perceptron. All the results derived in this paper can then be applied to a perceptron with transfer function 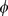, except that 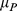 and 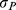 are now defined to be the two first moments of 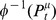.

### Storage capacity

In the large 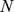 limit the probability of finding a set of weights that satisfies 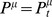 for all 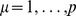 is expected to be 1 if 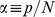 is below a critical value 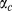, while it is 0 when 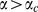 [15]. 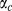 is therefore the number of associations that can be learned per synapse, and is commonly used as a measure of storage capacity.

This storage capacity can be computed analytically using the replica method (see Methods) [6], [16], [17], [10], [15]. The capacity is given by
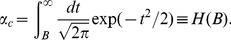
(2)

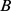
 is given by the equation
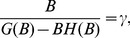
(3)

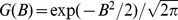
, 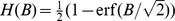, and 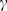 depends on the statistics of the associations as
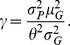
(4)


**Figure 1:**
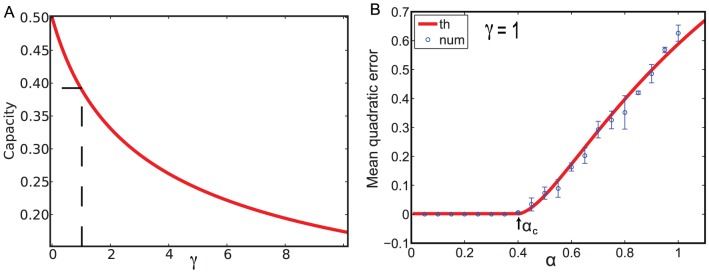
A. Maximal capacity as a function of 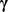. **B**. Mean squared error between the output 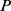 and the target output 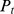 as a function of 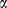, for 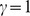 (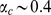). Red: analytical calculation, Eq. (17); Blue, numerical simulations (with parameters: 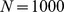, 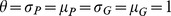, 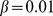, simulation length 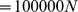, average over 20 trails, error bars: standard deviation).

Therefore, the maximal capacity only depends on a single parameter 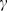, which is a function of the statistics of the patterns that need to be learned. This dependence is shown in Fig. 1A. It shows that the capacity is exactly equal to 0.5 when 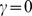, while it decreases monotonically as 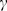 increases.

If the number of patterns to be learned exceeds the maximal capacity, the mean squared error becomes strictly positive. It can also be computed using the replica method (see Methods, Eq. (17)). Unsurprisingly, it increases monotonically with 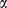, as shown in Fig. 1B which shows the result of the analytical calculation, as well as numerical simulations. If uncorrelated noise is added to the perceptron, the total mean squared error is the sum of the error without noise (Eq. 17) and the variance of the uncorrelated noise.

In the simulations, inputs and outputs are drawn from an exponential distribution. The weight update at each presentation is the standard perceptron one, i.e.
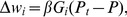
(5)where 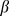 is the learning rate. 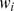 is set to zero if application of the update leads to a negative weight. This corresponds to a gradient descent of a cost function proportional to 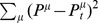, in the closed orthant 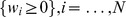.

This learning rule is in qualitative agreement with experimental data on synaptic plasticity in GC to PC synapses [18], . In Purkinje cells, the error signal is thought to be conveyed by climbing fiber (CF) activation. Two protocols have been shown to be effective in eliciting long-term plasticity. Pairing GC with and CF activation leads to Long-Term Depression (LTD) of the synapse, while Long-Term Potentiation (LTP) is induced by stimulating the GC alone (see Fig. 3AB of [19] for details). Writing climbing fiber activation as 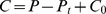, we see that Eq. (5) is recovered if one chooses 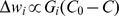, which captures the two experimental protocols described above.

### Distribution of synaptic weights

**Figure 2:**
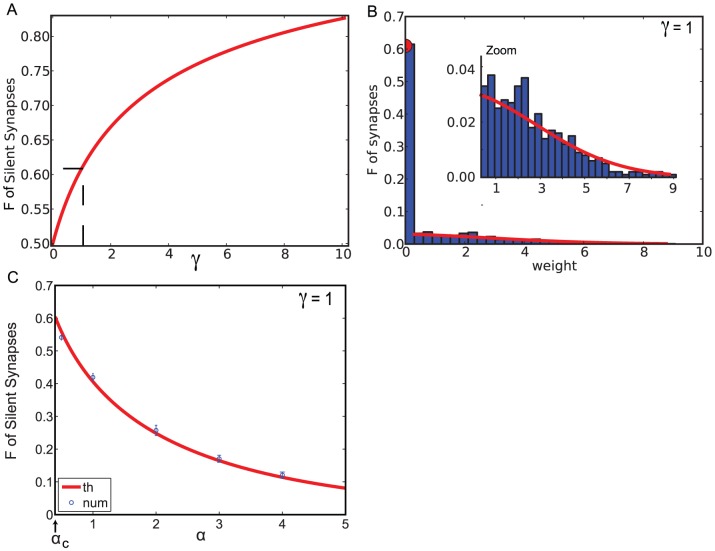
A. Fraction of silent synapses at maximal capacity as a function of 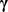. **B**. Distribution of synaptic weights for 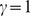, at maximal capacity (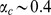). Red: analytical calculation, Eq. (6); Blue, numerical simulations (with parameters: 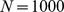, 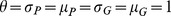, 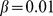, simulation length 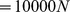). C. Fraction of silent synapses as a function of 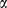, beyond the maximal capacity (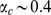), for 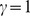 (red: analytical calculation, 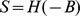); blue: numerical simulations, with parameters 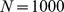, 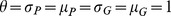, 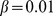, simulation length 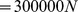, average over 10 trails, error bars: standard deviation).

The distribution of synaptic weights at maximal capacity can also be computed using the replica method (see [10] for details of the calculation). It turns out that the distribution obeys exactly the same equation as in the binary perceptron, i.e.
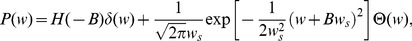
(6)where
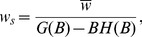
(7)and 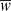 is the average synaptic weight. In particular the fraction of zero weight synapses is 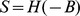. Interestingly, there is a very simple relationship between capacity and fraction of silent synapses, 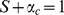, that holds for any value of 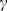. The fraction of silent synapses 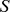 is shown as a function of 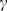 in Fig. 2A. It shows that 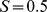 when 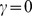, and increases monotonically with 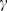.

The full distribution of weights is shown in Fig. 2B, together with the results of a numerical simulation (see parameters in the caption of Fig. 2B). The theoretical distribution of synaptic weights is in good agreement with experimental measurements of the efficacy of a large set of GC to PC synapses, using paired recordings in vitro (see Fig. 6A of [10] for details) [20], [21], [10].

Above maximal capacity, 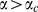, the distribution of synaptic weights is still given by Eq. (6), but the fraction of zero weight synapses decreases monotonically with 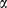, and goes to zero in the large 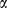 limit (see Fig. 2C). In that limit the distribution becomes increasingly close to a Gaussian distribution peaked around a positive value, with a width that tends to zero in the large 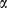 limit.

### Statistics of inputs and outputs maximizing storage capacity

To maximize storage capacity, 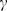 should be as small as possible. We first ask which distribution of inputs maximize capacity. From Eq. (4), it is clear that to maximize capacity, 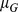 should be as small as possible, while 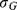 should be as large as possible. Since 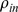 is a distribution of firing rates, it must be bounded between 0 and a maximal firing rate 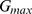. The distribution of a bounded variable that maximizes the variance with a fixed mean 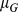 is a binary distribution 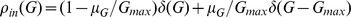. Thus, we predict that to optimize capacity, patterns of activity in the Granule cell layer should be sparse (to ensure 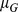 is small), but active cells should be active close to their maximal firing rates. Interestingly, this is in striking agreement with available data [22], [19], [23] showing that (i) Granule cells have very sparse activity in vivo (average firing rates of 0.5 Hz [22]) (ii) they can respond with brief, high frequency bursts of action potentials to sensory inputs (with an average frequency of 77 Hz within the burst, and maximal frequencies up to 250 Hz, see e.g. Fig. 3 of [22]).

**Figure 3:**
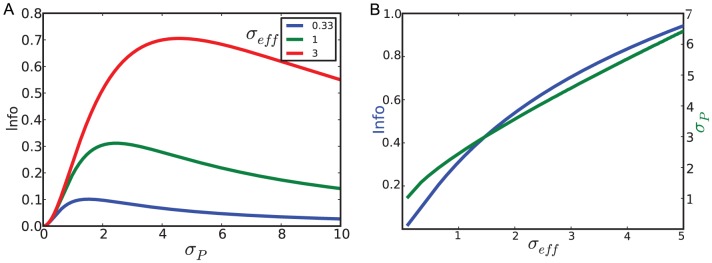
A. Information as a function of 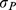 for different levels of 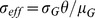, and 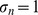. B. Optimal 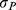 (green line, right y-axis) and information (blue line, left y-axis) as a function of 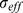.

The next question is which distribution of output firing rates optimizes the capacity. Eq. (4) makes it clear the capacity is optimized for 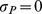. In this limit however, all input patterns lead to exactly the same output, and the Purkinje cell output contains no information on which input was presented. This is of course not a desirable outcome, and suggests the capacity is not the correct measure to maximize in this case. We therefore turn to the Shannon mutual information between the Purkinje cell output and its inputs as a more suitable measure. In the presence of additive Gaussian noise of zero mean and standard deviation 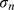, this is simply the mutual information of a Gaussian channel with a signal-to-noise ratio 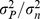, i.e. 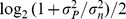 bits per pattern (see e.g. [24]). The total information in bits per synapse is therefore 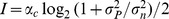. The information is zero when 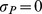, and reaches a maximum for a finite value of 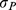, which depends on both the noise standard deviation 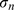 and 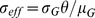. Fig. 3A shows the information as a function of 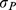, for different values of 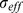, for 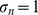. It shows that the optimal value of 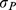 increases approximately linearly with 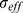 for large 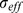 (see Fig. 3B).

## Discussion

In this paper, we have considered an analog firing rate model for a Purkinje cell with plastic excitatory weights, and derived both its maximal capacity and the distribution of weights at maximal capacity. We showed that the capacity is of the same order as in a binary perceptron model.

The distribution of synaptic weights of the analog perceptron is composed at maximal capacity of two parts: a large fraction (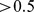) of silent synapses and a truncated Gaussian. It has exactly the same shape as in several other models: a standard binary perceptron [10], and a bistable perceptron [25]. This distribution is in quantitative agreement with a combination of electron microscopy and electrophysiological data in adult rat slices [20], [21], [10]. Furthermore, a gradient descent learning rule leading to maximal capacity bears strong similarities with synaptic plasticity experiments: LTD when PF and CF are coactivated, LTP when PF fires alone (i.e. CF below baseline, thus 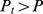) [18], [19].

We found that in order to maximize the capacity, the input variance should be as large as possible. We argue that GCs in vivo are close to such an optimal distribution, since they fire high-frequency bursts at very low rates [22], [19], [23]. Furthermore, GC bursts have been found in some experiments to be critical to induce plasticity in PF to PC synapse [26]. Indeed, no plasticity is induced in those protocols with a single GC spike. Secondly, lower variance in the output also increases the capacity, but at a cost of losing information contained in the output, in the presence of noise. For a given variance of the noise, there is an optimal variance of the output that maximizes the information contained in the output.

The model we have studied here is essentially equivalent to the ADALINE (Adaptive Linear Neuron) model [27], whose storage capacity, in the absence of constraints on synaptic weights, is equal to 1. The result can be easily intuitively understood by the fact that when 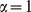, there are exactly N linear equations to solve, Eq. 1, with N unknowns, 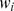 (see e.g. [15]). We have shown here that the constraints that all synaptic weights should be positive or zero leads to a capacity which is decreased by a factor 2 or more, depending on the value of 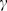. This decrease in capacity is similar to what is observed in the standard perceptron with excitatory synapses [7], [8], [9], [10]. Note that learning associations with constrained weights is similar conceptually to non-negative matrix factorization [28], [29]. Generalizations of such models in the temporal domain (the so-called adaptive filter models) have been proposed to describe learning in the cerebellar cortex [30], [31], [32], [33]. It would be of interest to investigate capacity and distribution of synaptic weights of such models.

In this paper, we have focused on a single plasticity site, the GC to PC synapse. Many other sites of plasticity are known to exist in the cerebellum [18]. Future studies are needed to clarify the impact of these additional sites of plasticity on the learning capabilities of this structure.

## Methods

### Calculation of the storage capacity

The replica method involves calculating the average logarithm of the volume of the space of weights satisfying all constraints given by Eq. (1) [6]. To compute the average logarithm, one uses the replica trick: 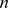 replicas of the system are introduced, one computes
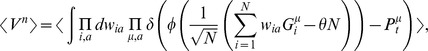
where 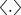 represents an average over the patterns, and 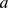 is a replica index. This calculation is done using a standard procedure. After introducing integral representations for the delta functions, one averages over the distribution of the patterns. One then introduces order parameters
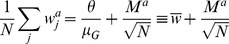
(8)

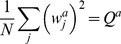
(9)

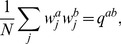
(10)together with conjugate parameters 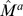, 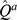 and 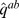. We then use a replica-symmetric ansatz (all the order parameters are taken to be independent of replica index 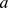), perform the limit 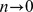 and obtain
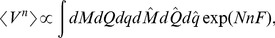
(11)

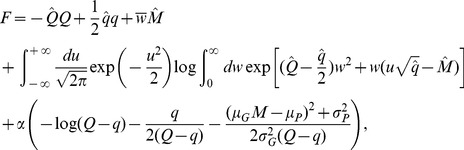
(12)where in the Equation for 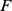 (Eq. (12)), the two first lines are identical to the binary perceptron with excitatory weights [10], while the last line is specific to the analog perceptron.

In the large 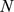 limit, the integral in Eq. (11) is dominated by the extremum of 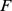. The typical values of all order parameters are then obtained by the resulting saddle point equations, setting the derivatives of 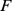 with respect to all order parameters to zero. The maximal capacity 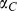 is obtained in the limit 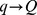, for which the volume vanishes. This limit yields Eqs. (2,4).

### Calculation of the mean squared error

Following [34], we introduce a cost function which is given by the sum of the squared error for all patterns,
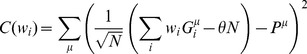
(13)and compute its minimum over the space of weights. This is done introducing a partition function 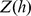,
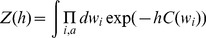
(14)where 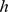 is an inverse temperature, and computing 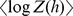 using the replica method. The mean squared error is then given by
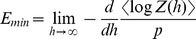
(15)


To perform this calculation, a new parameter has to be introduced,
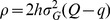
(16)which will remain finite when 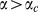 in the limit 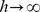, 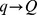. The mean squared error is then given by
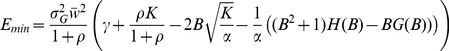
(17)where
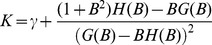
(18)

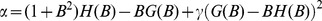
(19)

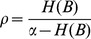
(20)When 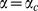, 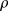 diverges to infinity, 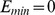, and Eqs. (19,20) reduce to Eqs. (2,3).
